# The Complexity of Crime Network Data: A Case Study of Its Consequences for Crime Control and the Study of Networks

**DOI:** 10.1371/journal.pone.0119309

**Published:** 2015-03-16

**Authors:** Amir Rostami, Hernan Mondani

**Affiliations:** Department of Sociology, Stockholm University, Stockholm, Sweden; Medical University of Vienna, AUSTRIA

## Abstract

The field of social network analysis has received increasing attention during the past decades and has been used to tackle a variety of research questions, from prevention of sexually transmitted diseases to humanitarian relief operations. In particular, social network analyses are becoming an important component in studies of criminal networks and in criminal intelligence analysis. At the same time, intelligence analyses and assessments have become a vital component of modern approaches in policing, with policy implications for crime prevention, especially in the fight against organized crime. In this study, we have a unique opportunity to examine one specific Swedish street gang with three different datasets. These datasets are the most common information sources in studies of criminal networks: intelligence, surveillance and co-offending data. We use the data sources to build networks, and compare them by computing distance, centrality, and clustering measures. This study shows the complexity factor by which different data sources about the same object of study have a fundamental impact on the results. The same individuals have different importance ranking depending on the dataset and measure. Consequently, the data source plays a vital role in grasping the complexity of the phenomenon under study. Researchers, policy makers, and practitioners should therefore pay greater attention to the biases affecting the sources of the analysis, and be cautious when drawing conclusions based on intelligence assessments and limited network data. This study contributes to strengthening social network analysis as a reliable tool for understanding and analyzing criminality and criminal networks.

## Introduction

Social network analysis (SNA) has been used to tackle a variety of research questions in different contexts. For example, its methods have been used to predict population displacement after natural disasters [1], to study disease control [2] and online social behavior [3]. In the case of criminal networks, the applications range from general criminality to terrorist networks, organized crime and street gangs. SNA has become an important method for understanding, assessing, and controlling crime networks and has implications for criminal policy. The value of SNA methods lies in their focus on the structure of the relationships in a network, rather than on the characteristics of individual actors [4]. SNA provides a framework for the abstraction and representation of a phenomenon in terms of interacting units and their relationships [5]. It comprises a set of methods and tools to gather, process, visualize, and model social network data [6]. In this context, network analysis has been a key tool of criminal intelligence analysis since the 1970s [7], from a basic technique in the form of link analysis in criminal intelligence and investigations, to the more recent implementation of core concepts and measures of social network analysis in problem solving strategies and criminal intelligence assessments e.g. [7,8]. Assessments of law enforcement serve as a guide in policymaking; consequently intelligence analysis based on SNA has implications for lawmaking and criminal policy. It is therefore crucial to better understand the complexity of different empirical data sources as they have implications for the results of network studies and assessments. Apart from the fact that crime networks are by definition difficult to access, the data on these networks are complex, to the extent that they come from different sources and suffer from different biases.

In this study we have the unique opportunity of having access to three different datasets on one specific Swedish street gang. These datasets are the most common information sources in studies of criminal networks, but it is uncommon that the data sources, intelligence, surveillance and co-offending data, are available for the same case. In this study we explore if different network datasets influence the results of criminal network studies and its consequences for intelligence assessments. We do it by building networks out of the datasets, and comparing them by computing distance, centrality, and clustering measures.

### Network concepts in a modern approach to policing

Law enforcement has special responsibilities like counterterrorism and controlling gangs and other crime networks [9–11]. The standard model of policing is a traditional reactive response to crime, random patrolling, crime investigations once an offence has been detected, and reliance on suppressive force and the legal system as the primary means of controlling and reducing crime [12,13]. However, policing in the last three decades have moved in the direction of problem-solving as a central strategy [14]. During the modern era of policing, several paradigms in police strategies have developed to curb the limitations of the standard model of policing, such as Community-Led Policing, Problem-Oriented Policing, Intelligence-Led Policing, and CompStat e.g. [14,15]. The essence of these strategies is to make policing more proactive in response to crime and to have police conduct special duties by acting on their own initiative, processing information about crime, and strategizing reduction and prevention [16]. One core element of these proactive strategies is based on crime and intelligence analysis, often based on SNA methods. Law enforcement collects or receives information on a problem they are trying to control, such as crime rates, other statistics, gang membership, terrorist organizations, crime networks, or an imminent crime etc. The intelligence is then assessed and serves to guide police management and operations in detecting, reducing, and disrupting of criminal activity, or solving a problem [15,17].

Social network analysis and intelligence-led policing have also gained popularity within the Scandinavian law enforcement and criminology community e.g. [18–23]. As such, the management of the Swedish police and all planned operations in the organization are based on PUM-*Polisens Underrättelsemodell*, a model inspired by The National Intelligence Model, which is a concept based on ideas drawn from intelligence-led policing e.g. [15,24]. The Swedish police, like many other law enforcement agencies, are paying more attention to intelligence assessments and as such to network analysis, for detecting, reducing, and disrupting the criminal activities of gangs and other networks [8]. In Denmark, law enforcement is using network analysis to detect gang members who may potentially be induced to leave their gangs [22]. As, such, network analysis is becoming an important component in both crime investigations and tactical and strategic intelligence assessments [7]. Together, crime intelligence has both a tactical and strategic role to play in policing, and constitutes a basis for planned operations and threat assessments for upcoming major events. Threat assessments by law enforcement agencies such as Europol, the Swedish Police, and the FBI are also used as a guide in policy-making. Therefore, intelligence analysis has implications for law-making and crime policy.

### Aim of this study

SNA has gained popularity in the study of social phenomena in general, and in studies of criminal networks in particular. It is furthermore becoming a key component of intelligence assessments. As more information is processed into the network framework, knowledge is accumulated and researchers will be able to provide better advice to policy makers. But this promising endeavor entails challenges as well. One of these challenges regards the use of different data sources to study the empirical phenomenon from a network perspective. This is a fundamental methodological issue within network science. However, this issue has not received enough attention in network studies, particularly in studies of crime networks. The aim of this study is to explore if different network datasets on the same phenomenon influence the results of criminal network studies, and its consequences for intelligence assessments. The methodological contribution of this paper is to use the case of a Swedish street gang to illustrate the complexity and reliability of empirical sources in analyzing criminal networks from a social network perspective.

## Materials and Methods

Our data for this study are extracted from the National Swedish Police Intelligence (NSPI), which has overall responsibility for collecting data on gang membership. Every regional and local crime intelligence unit in Sweden has the task of collecting information from police officers, units, and other relevant intelligence sources to identify gang membership and report this to NSPI [25]. NSPI then collects and registers the information.

When the Swedish police registers individuals as gang members, the member’s affiliation must be confirmed by different verified sources such as informants, undercover police, surveillance teams, telephone intercepts, open and other technical sources, or by eyewitness testimony from police officers where they have observed the individual carry signs and symbols of gang membership. When the information is assessed, the Swedish Crime Intelligence Unit in the region where the gang affiliation was observed registers the information in the Police Intelligence Registry (PIR), which consists of several different databases. This procedure is not formalized, but it is part of the overall guidelines for registering intelligence information by Swedish law enforcements e.g. [26].

We have used primary sources from the PIR to identify gang members in one specific street gang. By doing so, we created a dataset of 28 gang members. Once we had established the identity of the gang members and gathered criminal records, we summarized the information in a separate electronic dataset. The criminal record consists of members in the street gang under study who were suspected of one or more offences in Sweden between 1995–2010, and who were registered as such by the police. All data were anonymized before analysis, but in such a way that each individual was given a unique identification number. The gang under study is a Stockholm-based street gang with some concentration in southern parts of Stockholm County, in areas that can be described as marginalized suburbs of the Swedish capital. All gang members are male, with average age 22.8 years and mean age 22.5 years, with heterogeneous ethnic backgrounds. Gang members are engaged in "cafeteria style" delinquency with high levels of violence, thefts, robbery and drug-related crimes. This gang exhibits similar crime versatility pattern to its European counterparts [25].

### Data sources and networks for analysis

Crime network data in general have limitations and biases. One general limitation is that access to these data is restricted due to the sensitivity of the information, both with respect to doing research with hard-to-access populations and to gaining access to data from criminal justice organizations [27]. Another general limitation that crime network datasets have in common is that the interaction between the law enforcement and the observed population affects the network structure and dynamics. Arrests, incapacitation or intelligence leaks can have an effect on patterns of relationships in a crime network. The police can change the structure and membership of observed network through active surveillance and intervention, such as surveillance resulting in an arrest of an actor, or members of the criminal network may restructure interactions with their subordinates in order to obscure their own involvement. Another general limitation is that crime network data suffers from various selection biases. For example, reliance on official co-offending data often “misses data” [28]. This means that many crimes that were never discovered or reported are not covered by the data. Another issue with co-offending data is the “innocence factor”, meaning that a person who has been suspected or charged for a crime might in fact be innocent and been registered wrongly. Common-sense interpretations of phenomena can also be misleading, because what you think to be true may not be [29]. An additional bias arises because certain populations are at higher risk of detection and thus are registered more often [30]. All of these limitations and biases are increased when it comes to surveillance and intelligence data, because the data collection and registration process have a higher subjective component and are less regulated. In the case of intelligence data based on human intelligence (HUMINT) there is an availability bias, stemming from the fact that the official has access only to the restricted view provided by the data source. This can lead to a kind of anchoring bias, in which only a partial subset of a phenomenon is explored [31]. In the case of surveillance data, it is easy to look only at what is deemed interesting and give it priority—and report and record these accordingly. This introduces a sampling bias. Additionally, by working intensively following a certain group, there is a risk that your approach influences your view of other aspects of the phenomenon, the so-called halo effect [29]. Finally, your approach can result in stereotypical judgments that may not be representative of reality. Table 1 summarizes the characteristics of the datasets.

**Table 1:** Dataset characterization.

**Data source**	**Intelligence**	**Surveillance**	**Co-offending**
Biases	Availability	Sampling bias	Missing data
	Anchoring	Halo effect	Common sense bias
	Halo effect	Representativeness bias	Innocence factor
Network	Intelligence network (IN)	Surveillance network (SN)	Co-offending network (CN)
Nodes	Gang members	Gang members and associates	Gang members and co-offenders
Links	Intelligence assessment	Surveillance observations	Co-offending in crime

#### Intelligence-based data and intelligence-based network (IN)

As mentioned in the introduction, intelligence information is the basis of assessments. In major operations targeting specific crime groups and networks, the police surveillance, investigation, and intelligence branches work in close collaboration to collect intelligence in order to bring down the targeted gang. In some cases, a temporary special registry is created specifically for this operation, which is erased after the operation. In other cases, the regular registries are used instead in different operational analyses and support the surveillance and investigation teams in their work. In our case, no special registry was created to target the gang. The intelligence units used intelligence from PIR in their assessments, which served as a foundation for on-going operations and investigation. From all of these materials, we have extracted and mapped the gang’s organizational structure according to these assessments. In the process, we developed got two sets of intelligence assessments: the first set from 2006 until 2007, and the second set from 2007 to 2010.

**Fig 1:**
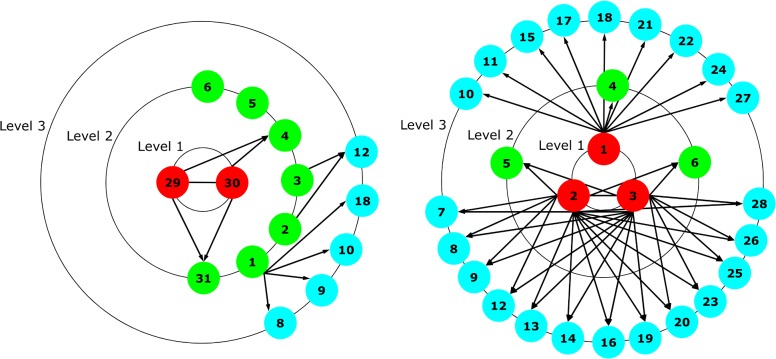
Intelligence-based networks (IN). Original gang hierarchy from intelligence analysis in 2007 (left) and hierarchy after intelligence update in 2010 (right). Nodes numbered by gang member number. The arrows illustrate the chain of command in the hierarchy.

Using these assessments, we plotted the gang’s organizational structure; one up to 2007 and the other up to 2010 (see Fig. 1 below). In these plots, links show the presumed lines of command, based on the police intelligence assessments. This constitutes a very basic technique in the form of link analysis, which is used in on-going operations and investigations by the police. Each gang member gets a gang member number that we will use to facilitate comparison and report our results. Additionally, a hierarchy number between 1 and 3, from the updated intelligence assessment, is given to all gang members. Data on the original hierarchy also come from the intelligence assessment, and so each gang member gets a hierarchy number between 1 and 3.

The plot in the left panel of Fig. 1 shows the IN from 2007. The intelligence assessment concluded that the street gang had 14 members arranged along three hierarchical levels. Two top leaders (gang members M29 and M30) constituted the highest level (1). The three top-level members acted as ‘Generals’ (according to the police intelligence, there were three generals, but according to the gang members themselves, the gang had only two Generals and one Sergeant-at-arms). Members M1-M6 and M31 were level 2 members, acting as so-called ‘captains’. The remainder of the members (M8-10, M12, M18) were level 3, so-called ‘Soldiers’. The plot in the right pane of Fig. 1 shows the same three hierarchical levels, but with a quite different allocation of members. As can be seen in the plot, the two individuals who were the top leaders in the IN of 2007 were expelled from the gang by the other gang members. But in the IN of 2010, we see that M4-M6 are still at level 2. Gang membership increased from 14 to 28 members in that period. Member M31 dropped out of the gang, and M1-M3 had assumed level 1 positions. In our intelligence-based material, we found that the police had neither calculated nor assessed this development. What we can conclude here is that the assessment from 2007 failed to predict this change and therefore we should also be critical about the assessment from 2010. One explanation for this could be that the police in these assessments used basic network techniques for understanding the gang structure before targeting it, rather than more sophisticated network measures that could be used for prediction of gang evolution. Note that the figure shows directed links just for illustration of the presumed command structure. In our study, networks are analyzed as undirected.

#### The General Surveillance Register and surveillance-based network (SN)

By law, the Swedish police also have the right to maintain a computerized General Surveillance Register (GSR) (Polisens allmänna spaningsregister, 2010:362). The purpose of the GSR is to maintain a general surveillance register regarding personal information revealed in law enforcement activities to facilitate access to the information needed in police operations. The GSR can maintain information about a person only if the person has already been convicted or suspected of a crime. The information should be relevant to police operations to merit registration. All police officers have access to the intelligence information in the GSR. As a result, the most sensitive intelligence information, including from human sources and telephone intercepts, is registered separately in other intelligence registries. After 2010, the Swedish police have been more inclined to open Special Intelligence Records or System (SUR/SUS), which are temporary. Unlike GSR, all intelligence is stored in these special registries. We have not used any information from these registries because our primary data collection was from before 2010.

The data from the GSR covers the period 1995–2010, and comes in the form of a link list. We first took out a few individuals who were not connected to others in the register. The link weight counts the number of occurrence of a given link in the list. The surveillance network was undirected as well. The nodes were, as before, assigned a gang member number and an updated hierarchy number. Three gang members were no longer part of the gang in the period covered by the data. We included them with their gang member number as isolated nodes to facilitate comparison between the three networks.

#### Co-offending data and co-offending-based network (CN)

We extracted co-offending data from governmental criminal records from 1995 to 2010, for all of the 28 gang members including the expelled gang members. Therefore, the total number of gang members in the study is 31. To be consistent and facilitate comparison, all 31 members are included in all of our networks.

We have information from the criminal records in the form of triplets (P_*i*_-C_*n*_-P_*j*_), meaning that person *i* is recorded on case *n* with person *j* as a co-offender. The first person in the recorded triplet (P*i*) is one of the gang members. A pair of individuals is considered only once per case. We build a node list with all people in the database (gang members and co-offenders) and relate gang members to the updated hierarchy from the intelligence assessment. Furthermore, we make the network undirected, so that the links (P_*i*_-P_*j*_), (P_*j*_-P_*i*_) are the same. Finally, we collapse the case variable to get a network of individuals. The final network is weighted, because a pair of individuals could be involved in more than one case. This weight is interesting for analytical purposes, since it gives a quantitative measure of activity between two individuals. Therefore, each computation takes the weight of the link into account.

### Network visualization, similarity, and measures

We analyze different network datasets of the same empirical phenomenon. In order to explore the complexity of the material, we conduct several comparisons of the different data sources. We compare the datasets using three techniques: network visualization, network similarity, and network measures. Network visualization means plotting the different network structures in a way that facilitates the understanding of structural differences and the comparison of properties across networks.

Network similarity, generally speaking, aims at developing methods to quantify how similar (or dissimilar) two given networks are [32]. Here, we use a very elementary measure, the *graph edit distance* (GED) as a first approximation of similarity [33].This distance between two given networks measures the number of node/link additions/deletions one has to perform to turn one network into the other, relative to the maximum number of changes. The larger distance, the more dissimilar the given networks are. The measure goes from 0 in the case were the networks are identical, to 1 when they are completely dissimilar.

Finally, let us introduce the network measures we use. The characterization of a social network, in particular of a criminal network, depends on a number of factors such as the research question and whether the data is dynamic or static, etc. Our aim in this paper is to provide a comparison of different network datasets constructed to study the same phenomenon. For this first research step, we want to keep the comparison as simple as possible, yet make it meaningful. We therefore choose three elementary measures for static undirected networks: two of centrality and one of clustering.

Actors interacting in a social group bring along different personal characteristics, they may perform specific activities, and have relationships with certain others. When looking at the relationships as a whole in a network framework, it is possible that a combination of the mentioned factors make certain actors more salient or important than others. Centrality measures in network analysis intend to give information of how important an actor is in a given network context. There are several ways to address this question. The *degree centrality* (*Dc*) of a node *i* [34] is simply the proportion of nodes that are connected to node *i*. This gives a rough measure of the activity/popularity of that node, assuming that the relevant interactions can be captured by the links. Our second centrality measure is the *betweenness centrality* (*Bc*) [35]. It is computed as the number of minimum paths between all pairs of nodes that pass through node *i*, as a fraction of all possible minimum paths. While degree centrality focuses on activity level via the number of links, betweenness centrality looks at the transfer of information or resources through a given node. Thus, it is a proxy for the brokerage capabilities of node *i*, in the sense of being able to bridge different parts of the network. It is important to have more than one centrality measure, as there could be a trade-off between being highly connected but more visible and thus more exposed (higher degree centrality) and being less visible but more instrumental in transmitting information between different network subgroups (higher betweenness centrality) [36]. Finally, we measure the *local clustering coefficient* (*C*_*i*_) of node *i* as the ratio between the number of pairs of adjacent nodes to *i* that are connected themselves, to the number of pairs of nodes that are adjacent to *i* (Newman 2010). This is a transitivity measure, giving us a quantitative idea of how likely it is that two people acquainted with *i* are also acquainted with each other. (We will use *C* for the local clustering coefficient for notation simplicity, but this should not be confused with the global clustering coefficient for the whole network.) High clustering may indicate a stronger likelihood to cooperate and build small operative subgroups. The measures for betweenness and clustering take into account the weight of the link.

We use the free software g*ephi* for network comparison [37]. Each node is assigned a position, so that nodes occurring in more than one network get the same position and can be compared easily. The color scheme is as follows: white for non-gang members, and colored for gang members, according to the updated hierarchy. Gang members are shown with their gang member numbers as well. We computed the network measures with the free package *networkx*, a python package for the analysis of complex networks [38].

### Comparison methodology

In practice, each policing analysis begins with an intelligence assessment of some kind. These assessments trigger other processes and have vast implications. Therefore, it is important to compare the intelligence networks with other available networks. Having access to such unique data makes it possible to perform relevant comparisons to contribute to refining SNA methods and understanding the complexity. Therefore, we compare the surveillance network with the co-offending network with respect to graph similarity as well as the three network measures. And finally, we explore the consequences of using all of the available data by merging the SN and CN and computing the network measures in this case.

## Results

### Comparing the IN with the other networks

#### IN 2010 vs. CN 2010

**Fig 2:**
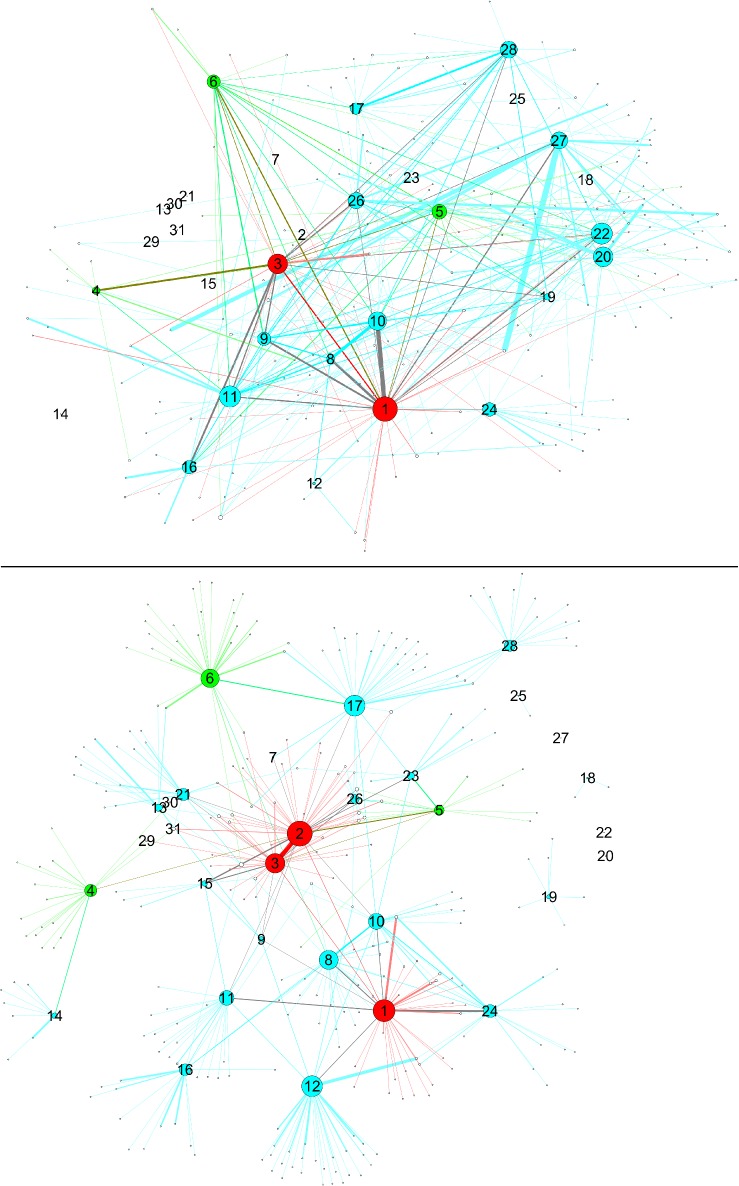
Networks with nodes sized by degree centrality. Upper panel: SN-surveillance network, lower panel: CN-co-offending network. Data from 1995–2010. Color scheme according to updated hierarchy.

The intelligence network (IN) and co-offending network (CN) may be similar on a first impression, in the sense that the top leaders have a central position in the visualization. Looking at the intelligence network from the 2010 assessment (IN 2010), Fig. 1, we see that members M1-M3 that are allocated in level 1 positions. However, the assessment concluded that the network was divided into two subgroups, with M1 at the head of one subgroup, and M2-M3 at the head of the other group. The co-offending data (see Fig. 2, lower panel) shows that the two subgroups are not actually disconnected, even if interaction seems to be more intense within the subgroups than between them. This may reflect the availability bias, where the assessment made inferences about the hierarchical structure of the gang without having the full picture.

Another thing to note is that level 2 members are more peripheral than level 3 members. One could assume that level 2 members do not directly take part in criminal activities, their positions being one of planning instead. But this does not hold, because we see that level 1 members are directly involved in co-offending links to an even higher degree than level 2 members. This may be an example of anchoring: the investigators assume the command structure to be central to the functioning of the gang, and by focusing on that aspect do not gain access to the more complex everyday reality. Finally, we can say that the intelligence assessment was slightly improved by detecting the true top leaders of the gang, but could not describe adequately the gang structure.

#### IN 2010 vs. SN

In this comparison, too, we see major differences between both intelligence networks. First, member M2, who had a top position in IN 2010, had no links at all in the SN. This means that if only the GSR data were available, we could conclude wrongly that this person has a very minor or even non-existent role in the gang. A representativeness bias is at play here.

Second, we cannot find in the SN a clear hierarchical pattern emerging just from the structure. It seems rather that the level 3 members occupy more central positions. This can be interpreted as the result of two sampling biases by police operations: i) high-ranked members are followed more because they are considered important; and ii) low-ranking members are often just observed because of their number and their salience. This can give a distorted picture of the situation. An element of the halo effect is also present in the fact that assuming that a high-level member is important to the command translates into thinking he is important in all activities, causing that member to be followed more often. Therefore, it seems that GSR data reflects more the nature of policing operations rather than the gang’s structure and dynamics.

#### IN 2007 vs. CN 2007

**Fig 3:**
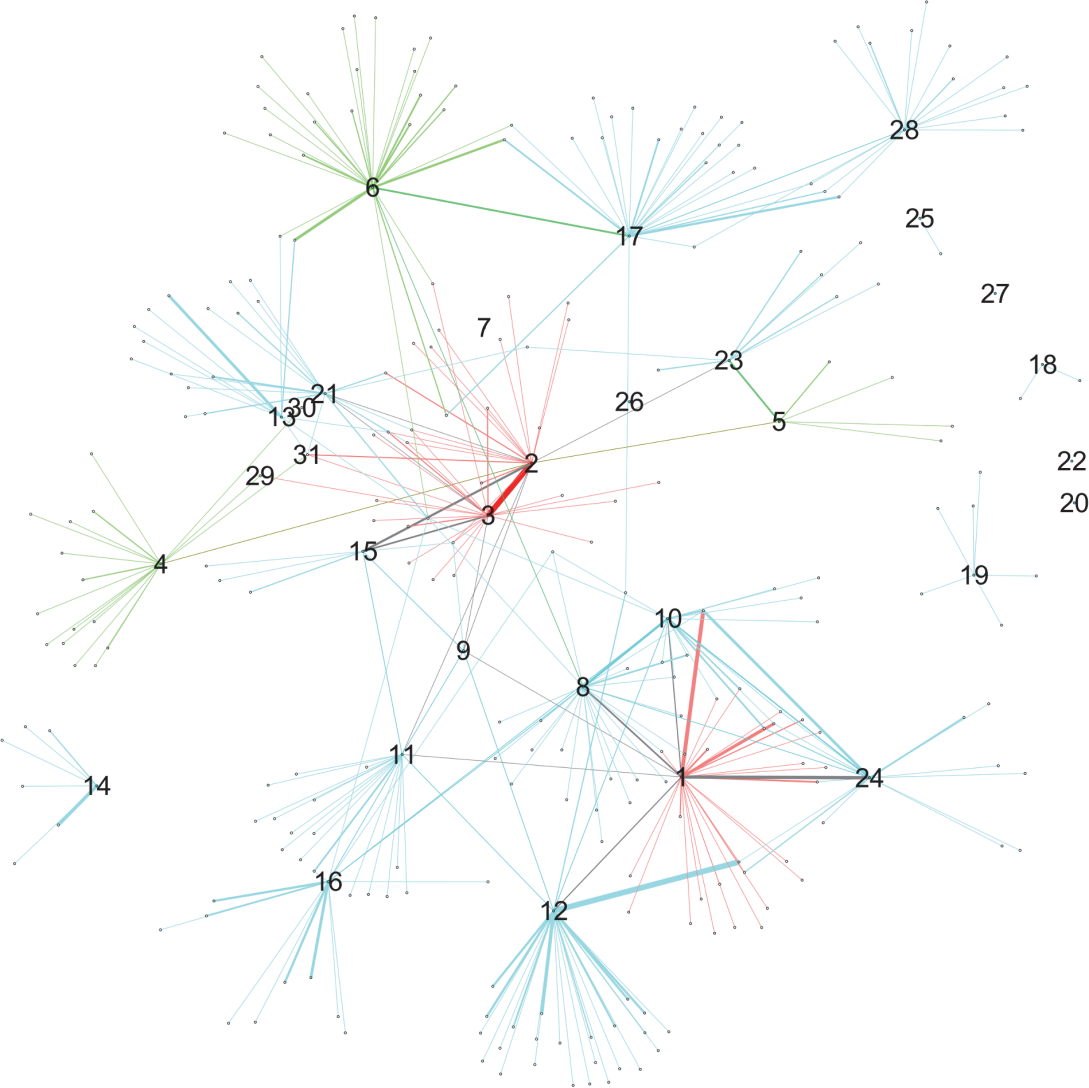
Co-offending network (CN) accumulated up to 2007.

One argument against the previous comments could be that the situation in 2010 is not a good starting point, because both the IN and the CN had already evolved over time. Therefore, we complement the analysis by looking at the situation back in 2007. We show in Fig. 3 the co-offending network up to the year 2007. In this comparison, a first observation is that the ‘President’ (M29) only has two actual links to other members. His position is thus quite peripheral, and this could possibly explain his eventual expulsion from the gang, despite his having been the founder. This was not what the assessment concluded; rather they assumed that he played a central role and controlled the gang. Another thing to notice is that the IN from 2007 concluded that the command structure in this street gang goes by levels, that is, that level 1 members control level 2 members, and level 2 members in turn control level 3 members. We can see that the co-offending data shows a different picture, with members from level 1 directly linked to level 3 members. We also see that the crime activity, understood as the number of co-offending links, is broadly distributed across members, with some having very few links and other many. However, this distribution does not match the hierarchy assessment in IN, since some members from all levels have a high link count. Consequently, we see that, even at this early stage, the assessment and the co-offending data give us different pictures of the situation.

### Comparing SN and CN

**Fig 4:**
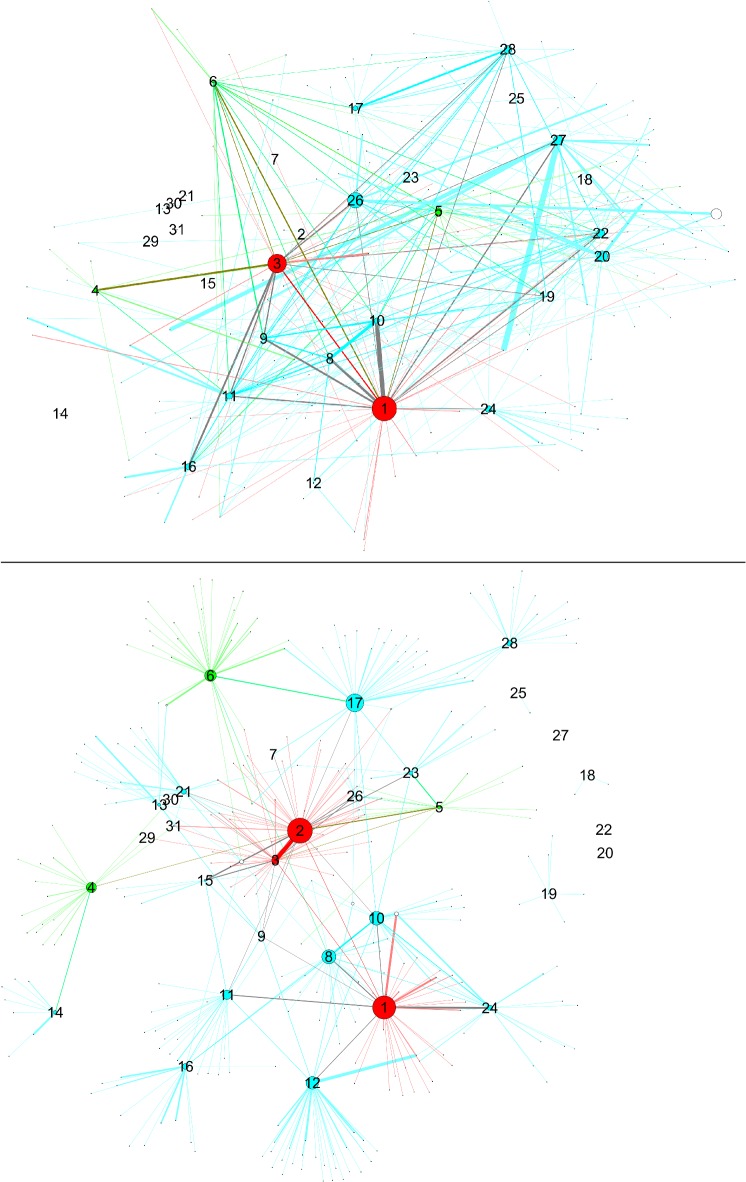
Networks with nodes sized by betweenness centrality. Upper panel: SN-: surveillance network, lower panel: CN: -co-offending network. Data from 1995–2010.

**Fig 5:**
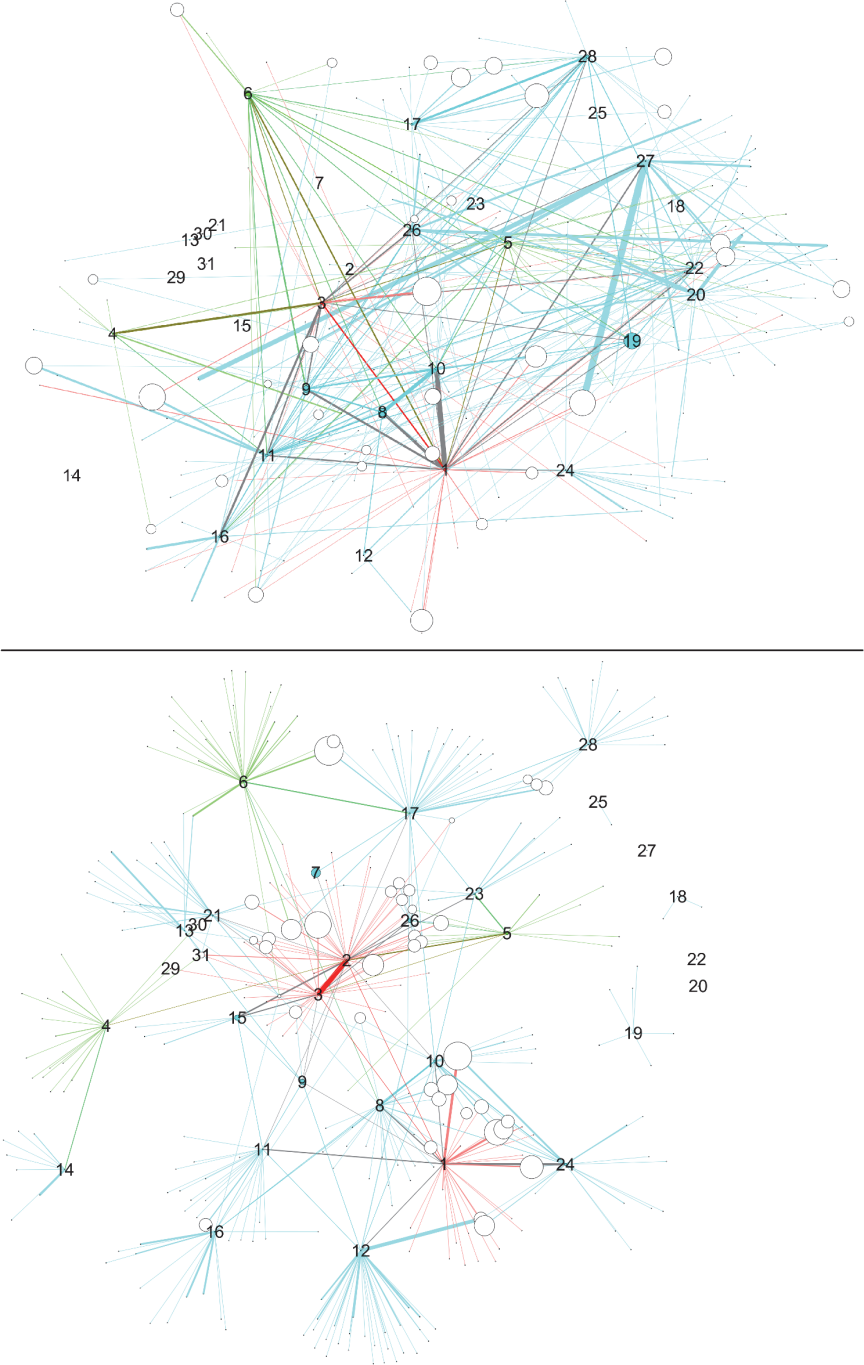
Networks with nodes sized by local clustering coefficient. Upper panel: SN-: surveillance network, lower panel: CN: -co-offending network. Data from 1995–2010.

In this comparison, we explore the SN and CN network structures, first by visualizing them, and second by analyzing their similarity and network measures. The network visualizations are shown in Figs. 3–5. Node sizes are proportional to the three network measures (degree centrality, betweenness centrality, and local clustering coefficient). In order to make it possible to display all of these combinations in a simple but meaningful way, we have one figure per network measure. There are two panes in each figure corresponding to the network datasets. As an overall comment, we can say that the two networks look qualitatively very different from each other in terms of number of nodes and connectivity. Depending on which source of data we compare, the picture of the situation can change quite a bit.

Now we make a comparison based on network similarity. The surveillance network SN has 234 nodes and 315 links, while the co-offending network CN has 311 nodes and 409 links. Given that both datasets come from the same street gang, a natural question to ask is to what extend the two networks differ in a meaningful way. To address this question, we use the notion of graph edit distance (GED). In our case, the calculation is simplified considerably by the fact that node labels are unique, so we can identify the overlap between SN and CN, which consists of 64 nodes. So, for instance, going from CN to SN implies 311–64 = 247 node deletions, and 234–64 = 170 node additions, for a total of 417 node changes. The link changes amount to 628, giving a GED of 1,045. The same is true for the inverse problem. The maximum number of changes would correspond to the case where the networks were completely different. In that situation, and taking CN as the start network, we would have to remove all of the CN (311 nodes and 409 links) and put all of the SN (234 nodes and 315 links), for a total of 1,269 changes. Therefore, the relative GED in our case is 1,045/1,269 = 0.82. This is clearly an indication that the networks are highly dissimilar, as the number is close to 1.

Going into network measures, Fig. 2 features node sizes by degree centrality. Level 1 and level 3 nodes are more central than level 2 nodes. As previous comparisons showed, the structures are quite different. The CN shows a clearer picture in terms of structuring and crime activities. The activities captured by the surveillance operation and represented in the SN do not clearly match the resulting criminal activities in the CN.

The case of betweenness centrality is seen in Fig. 4. Here, the highest centrality scores do correspond to gang members. On the other hand, it is interesting to observe the interaction between betweenness centrality and hierarchy. In the surveillance and especially in the co-offending network, level 1 nodes (i.e., gang members 1–3) display high centrality scores, indicating that positions up in the hierarchy have higher brokerage potential. However, nodes having scores next to the highest ones do not belong to level 2, but to level 3 (gang members M7 and up). So the hierarchy does not reflect the ordering coming from betweenness centrality scores.

Finally, Fig. 5 presents nodes scaled by local clustering coefficient. One interesting feature of this plot is that nodes with high values of local clustering are, for the most part, not gang members. Those gang members who have high clustering differ across networks. This pattern applies to both networks and suggests that processes of cooperation and crime co-offending surpass gang boundaries to a certain extent. Moreover, in the CN, one high-ranked node is M31, who is actually one of the members that was expelled from the gang.

A summary of network properties and measures is shown in Tables 2 and 3. The full numerical results are reported in Table 4. Table 4 features the three network measures for each of the network datasets. Scores are shown by gang member number, accompanied by the ranking in each list. Low rank numbers correspond to high values on that particular list. Lists are ordered by the rank number in the co-offending network. The top five scores are shown in bold numbers. Hierarchy ranking according to IN 2007 and 2010 are shown for reference and comparison.

**Table 2:** Summary of network properties, by network dataset.

**Property**	**SN**	**CN**	**SN+CN**
Number of nodes	234	311	481
Number of links	315	409	676
Number of components	13	7	3
Density	0.012	0.008	0.006

**Table 3:** Summary of network measures, by network dataset.

**Statistic**	**Dc**			**Bc**			**C**		
	**SN**	**SN+CN**	**CN**	**SN**	**SN+CN**	**CN**	**SN**	**SN+CN**	**CN**
Mean	0.0523	0.0524	0.0484	0.0781	0.0922	0.0783	0.0129	0.0141	0.0106
Median	0.0472	0.0604	0.0419	0.0421	0.0791	0.0425	0.0044	0.0082	0.0030
Std. dev.	0.0492	0.0345	0.0390	0.1049	0.1169	0.0947	0.0231	0.0202	0.0169
Range	0–0.15	0.01–0.13	0–0.13	0–0.44	0–0.52	0–0.35	0–0.12	0–0.1	0–0.08

**Table 4:** Network measures and ranking, by network dataset.

**Dc**							**Bc**							**C**						
**Node**	**SN**	**r**	**SN+CN**	**r**	**CN**	**r**	**Node**	**SN**	**r**	**SN+CN**	**r**	**CN**	**r**	**Node**	**SN**	**r**	**SN+CN**	**r**	**CN**	**r**
2	0.000	14	0.083	7	0.129	1	2	0.000	18	0.106	10	0.345	1	7	0.000	18	0.053	2	0.071	1
1	0.146	1	0.123	1	0.113	2	1	0.434	1	0.518	1	0.318	2	31	0.000	18	0.044	3	0.047	2
17	0.064	9	0.085	6	0.106	3	17	0.078	12	0.114	5	0.242	3	9	0.044	3	0.036	6	0.041	3
12	0.013	13	0.073	9	0.106	3	8	0.033	16	0.077	17	0.190	4	15	0.000	18	0.036	5	0.039	4
3	0.116	3	0.110	2	0.100	4	10	0.086	11	0.079	16	0.184	5	26	0.015	10	0.012	8	0.028	5
8	0.047	10	0.073	9	0.097	5	12	0.009	17	0.112	6	0.167	6	5	0.004	16	0.006	18	0.018	6
6	0.077	8	0.088	5	0.094	6	6	0.042	15	0.182	3	0.155	7	24	0.001	17	0.005	19	0.014	7
10	0.107	4	0.094	4	0.081	7	4	0.059	13	0.110	7	0.135	8	10	0.020	5	0.012	7	0.012	8
11	0.124	2	0.098	3	0.074	8	11	0.104	9	0.109	8	0.120	9	3	0.018	8	0.009	14	0.010	9
24	0.077	8	0.069	10	0.065	9	3	0.326	2	0.475	2	0.093	10	2	0.000	18	0.009	13	0.009	10
4	0.043	11	0.060	11	0.061	10	16	0.098	10	0.093	13	0.072	11	23	0.000	18	0.008	17	0.008	11
21	0.000	14	0.040	14	0.061	10	28	0.128	7	0.109	9	0.072	12	1	0.018	7	0.010	11	0.007	12
16	0.077	8	0.069	10	0.058	11	24	0.111	8	0.094	12	0.066	13	21	0.000	18	0.004	21	0.005	13
28	0.099	5	0.075	8	0.055	12	21	0.000	18	0.041	20	0.064	14	8	0.057	2	0.011	10	0.005	14
5	0.086	7	0.069	10	0.048	13	23	0.000	18	0.028	22	0.049	15	16	0.006	13	0.004	22	0.003	15
26	0.094	6	0.069	10	0.042	14	14	0.000	18	0.029	21	0.043	16	11	0.016	9	0.008	15	0.003	16
13	0.000	14	0.023	16	0.035	15	5	0.111	8	0.080	15	0.037	17	28	0.019	6	0.009	12	0.003	17
23	0.000	14	0.023	16	0.035	15	13	0.000	18	0.021	24	0.033	18	17	0.010	12	0.003	23	0.002	18
9	0.077	8	0.048	13	0.026	16	15	0.000	18	0.012	25	0.019	19	6	0.038	4	0.008	16	0.001	19
14	0.000	14	0.017	17	0.026	16	9	0.051	14	0.043	19	0.011	20	12	0.000	18	0.003	24	0.001	20
15	0.000	14	0.017	17	0.026	16	31	0.000	18	0.000	27	0.011	21	4	0.006	14	0.002	25	0.001	21
19	0.034	12	0.029	15	0.019	17	26	0.270	3	0.153	4	0.001	22	29	0.000	18	0.096	1	0.000	22
31	0.000	14	0.010	18	0.016	18	19	0.000	18	0.025	23	0.000	23	19	0.114	1	0.036	4	0.000	22
7	0.000	14	0.004	19	0.006	19	18	0.000	18	0.000	26	0.000	24	22	0.011	11	0.011	9	0.000	22
18	0.000	14	0.004	19	0.006	19	20	0.186	4	0.097	11	0.000	25	27	0.005	15	0.005	20	0.000	22
29	0.000	14	0.004	19	0.006	19	27	0.162	5	0.081	14	0.000	25	13	0.000	18	0.000	26	0.000	22
25	0.000	14	0.002	20	0.003	20	22	0.136	6	0.069	18	0.000	25	14	0.000	18	0.000	26	0.000	22
30	0.000	14	0.002	20	0.003	20	7	0.000	18	0.000	27	0.000	25	18	0.000	18	0.000	26	0.000	22
22	0.124	2	0.060	11	0.000	21	25	0.000	18	0.000	27	0.000	25	20	0.000	18	0.000	26	0.000	22
20	0.116	3	0.056	12	0.000	21	29	0.000	18	0.000	27	0.000	25	25	0.000	18	0.000	26	0.000	22
27	0.099	5	0.048	13	0.000	21	30	0.000	18	0.000	27	0.000	25	30	0.000	18	0.000	26	0.000	22

Tables sorted by CN ranking.

### Merging SN and CN

**Fig 6:**
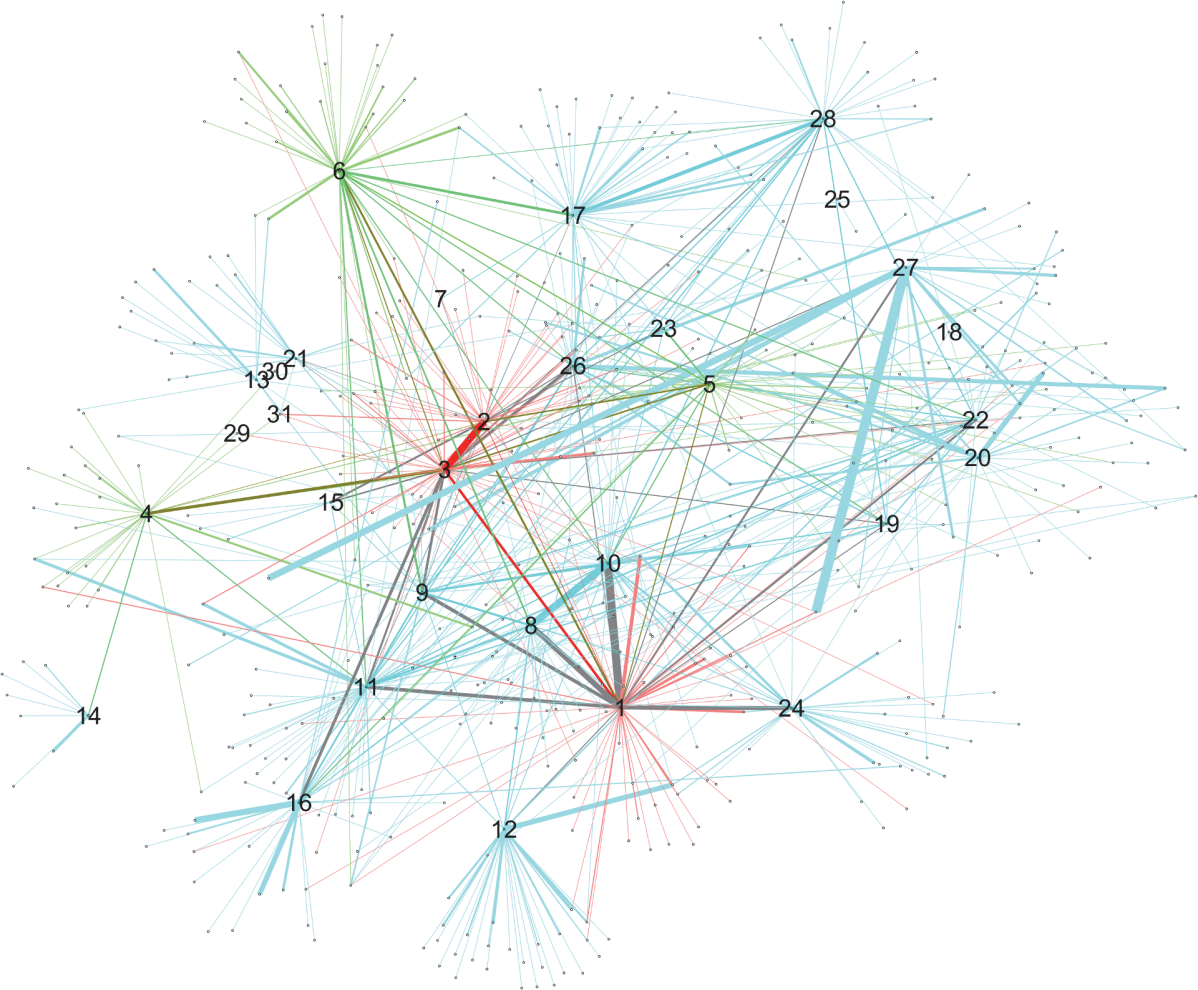
Merging of SN and CN networks. Data from 1995–2010.

One natural thing to do when one has more than one data source is to check what happens when they are combined into one network. The resulting network is shown in Fig. 6. Network measures are displayed in Table 4 along with the measures for SN and CN. It is perhaps not surprising that the merged network gives an intermediate situation between CN and SN. However, overall it is closer to CN in its statistical features, although the rankings differ.

**Fig 7:**
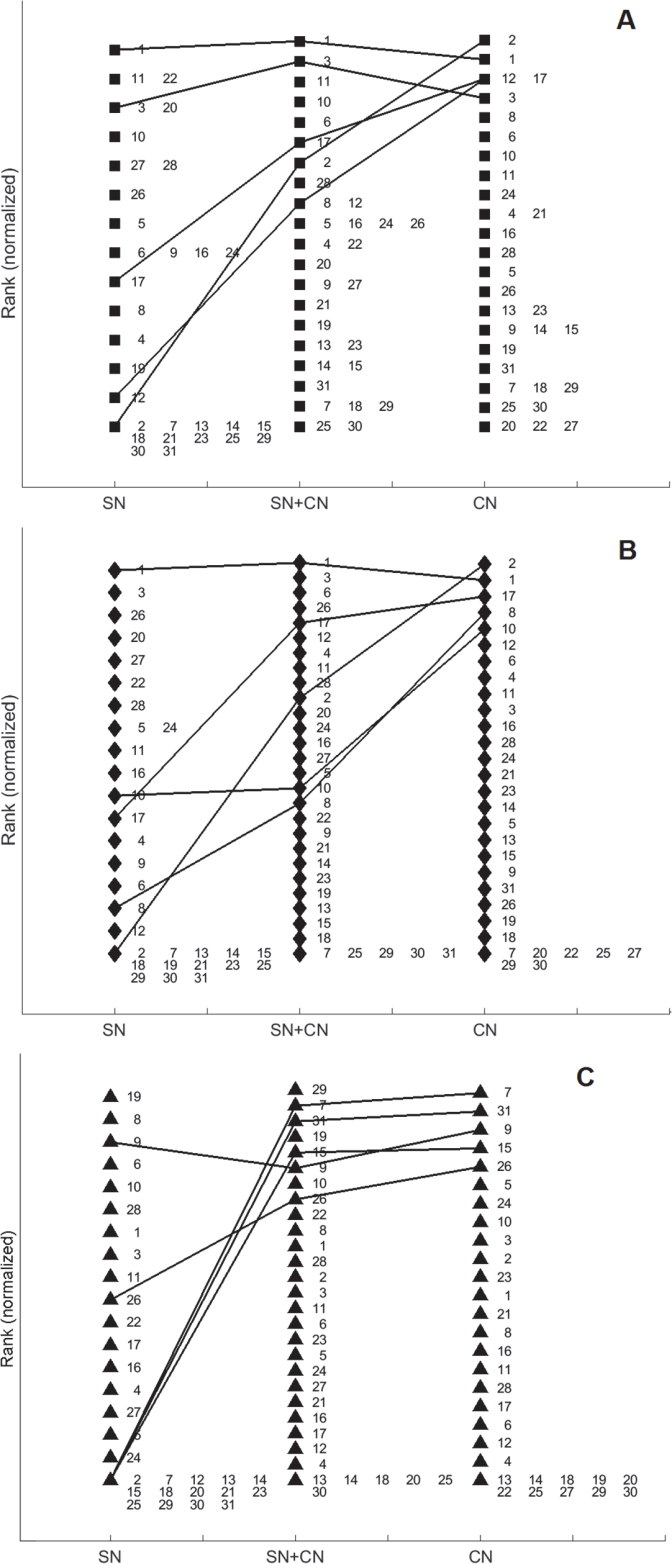
Normalized rankings across networks, by: (A) degree centrality, (B) betweenness centrality, and (C) local clustering coefficient. Coloring according to updated assessment: red = level 1, green = level 2, blue = level 3, white = former member (color combinations are shown were more than one hierarchy level is present).

As a visual summary and complement, we plot in Fig. 7 the ranking variation across networks, for each network measure. Each rank is normalized by the maximum value on that list, so that it goes from 0 to 1, that is, from high values to low values. A line shows the ranking of the five nodes with highest rank in the CN as reference, in order to show how the most important nodes are ranked in different networks and when using different measures. We can use this view to further strengthen our previous comments. The ranking patterns are different in all combinations. They differ not only when changing the network measure, but they display very different rankings across networks for the same measure. Overall, the SN tends to invert some of the top five positions, assigning low rank to nodes having high rank in the other two networks.

We further compute the Pearson correlation coefficient between the network measures for each pair of datasets (see Table 5). As can be seen in the table, the measures are weakly correlated across networks, even slightly anticorrelated in the case of the local clustering. Only when adding the merged network the correlation increases, by virtue of duplicated information, of course.

**Table 5:** Pearson correlation between network measures.

**Combination**	**Dc**	**Bc**	**C**
SN—CN	0.27	0.25	-0.08
SN—(SN+CN)	0.78	0.86	0.15
(SN+CN)—CN	0.80	0.54	0.48

## Discussion

SNA techniques have improved and become more sophisticated. However, social behavior is as complex as ever, and the different data sources reflect this complexity. Studies and assessments of criminal networks need to take into account the “complexity factor”. Network datasets are complex, both because of the nature of network data but also because of their particular biases and limitations. The aim of this study was to explore whether different datasets influence the results of network analyses. Our results show clearly that different datasets for the same phenomenon produce fundamentally different pictures. The same individuals have different importance ranking depending on the dataset and network measure with implications on crime control. For example the question of whom to target in a planned operations becomes not easy to answer. Consequently, using different datasets can have implications for the results of crime and network studies as well as intelligence assessments.

Our comparison shows that intelligence-based networks are substantially different from both surveillance and co-offending networks. This is an indication that the view presented by police assessments can greatly distort the actual facts, and that they suffer from availability, anchoring, and halo effect biases. Significantly though, all policing decisions are made based on these assessments. It is of course necessary to discuss the extent to which the SN and CN data reflect accurately the empirical reality of our case study. We cannot claim any definitive conclusion here, because that reality is ultimately accessible only by gathering data. What we can say is that a clear selection bias impacts the surveillance network in particular, since people deemed more central are followed more often than others. This situation is further complicated by a representative bias. The co-offending data, on the other hand, is based on actual crime instances, so the data is more objective in that sense, and would presumably be closer to the actual facts. However, co-offending dynamics suffer from various biases as well, such as missing data, the “innocence factor,” and the common sense bias, and they may be just one of many aspects driving gang formation and evolution, so the CN data is still limited. The same goes for the surveillance network as well as the merged network. The dataset greatly affects the results of network analyses.

The results of our study have implications for studies of crime networks and crime control. Crime-fighting authorities have moved toward more proactive strategies for combating crime, gangs, and terrorism. Within these strategies, intelligence analyses and assessments play a vital role. Intelligence assessments by law enforcement are having a great impact on policymaking. These two fields, SNA and intelligence-led crime prevention, have influenced each other: to the extent that SNA is becoming a core component of intelligence assessment (and to some degree of crime prevention) but also to the extent that researchers are using all available data to understand delinquency and human behavior. Our study has shown, the intelligence assessment based on limited network data should be viewed critically, and strengthened by the use of other data sources. Researchers, policy makers, law-enforcement officers, and other practitioners need to take the qualitative understanding of crime networks into consideration. Furthermore, they need to rely on data sources having fewer selection biases, such as co-offending, and at the same use multiple data sources to detect key features in the targeted phenomenon. The same goes for research on crime and delinquency.

Our aim is not to criticize or undermine social network studies, but rather to illustrate the complexity of the problem, and to contribute to more reliable results. One possible strategy to deal with the complexity of data and limit particular biases is to complement the analysis with a different approach such as by gathering qualitative data from interviews with members of the network under study to capture the essence of the network structure.

The strength that has been put into refining the methods of networks analysis should also be directed toward understanding the complexity of network data sources. Further research should look into other cases with multiple data sources. Our intention is to continue studying criminal networks through different lenses.
